# A newly developed kit for dental apical root resorption detection: efficacy and acceptability

**DOI:** 10.1186/s12903-024-04056-5

**Published:** 2024-03-02

**Authors:** Jun Hong Steven Tan, Farinawati Yazid, Nurfathiha Abu Kasim, Shahrul Hisham Zainal Ariffin, Rohaya Megat Abdul Wahab

**Affiliations:** 1 Department of Family Oral Health, Faculty of Dentistry, Universiti Kebangsaan Malaysia, Jalan Raja Muda Abdul Aziz, Kuala Lumpur, 50300 Malaysia; 2 Halvec Laboratories Sdn. Bhd, G-E-4, Enterprise 4, Technology Park Malaysia, Bukit Jalil, Kuala Lumpur, 57000 GF Malaysia; 3 Department of Biosciences and Biotechnology, Faculty of Science, Universiti Kebangsaan Malaysia, Bangi, 43600 Selangor Malaysia

**Keywords:** Orthodontic, Root resorption, Biomarker, Gingival crevicular fluid, Dentine sialophosphoprotein

## Abstract

**Objectives:**

To determine the efficacy of a newly developed kit in dentine sialophosphoprotein (DSPP) detection and compare it with enzyme-linked immunosorbent assay (ELISA). User acceptance was also determined.

**Materials and methods:**

This cross-sectional study consisted of 45 subjects who were divided into 3 groups based on the severity of root resorption using radiographs: normal (RO), mild (RM), and severe (RS). DSPP in GCF samples was analyzed using both methods. Questionnaires were distributed to 30 orthodontists to evaluate future user acceptance.

**Results:**

The sensitivity and specificity of the kit were 0.98 and 0.8 respectively. The DSPP concentrations measured using ELISA were the highest in the RS group (6.33 ± 0.85 ng/mL) followed by RM group (3.77 ± 0.36 ng/mL) and the RO group had the lowest concentration (2.23 ± 0.55 ng/mL). The new kit portrayed similar results as the ELISA, the optical density (OD) values were the highest in the RS group (0.62 ± 0.10) followed by RM group (0.33 ± 0.03) and the RO group (0.19 ± 0.06). The differences among all the groups were statistically significant (*p* < 0.05) for both methods. The Pearson correlation coefficient showed a statistically significant (*p* < 0.001) strong and positive correlation between DSPP concentrations and OD values.

**Conclusions:**

The new kit was validated to detect the colour intensities of different severity of root resorptions. Most of the responses to the survey were positive towards the new kit for being a safer and simpler method to detect apical root resorption.

## Introduction

Apical root resorption is a common and undesirable sequela of orthodontic tooth movement, it is also known as orthodontically induced inflammatory root resorption (OIIRR). The prevalence of OIIRR ranges from 44 to 91% according to various reported studies [1]. Most patients experience mild to moderate root resorption after orthodontic treatment which is clinically insignificant and does not compromise their dentitions. However, some patients may experience severe root resorption, resulting in tooth mobility and could potentially jeopardize the success of orthodontic treatment [1–3].

Currently, the clinical diagnosis of root resorption is mostly obtained through radiographic examination. But the downside of this method is it is technique sensitive, hard to achieve standardization, and requires repetitive radiation exposure to monitor the progression of root resorption. Radiographic examination is also unable to accurately identify root resorption at an early stage as it can detect root resorption only after approximately 60–70% of mineralized tissue loss and cannot show if the process of root resorption is still active [4]. Early detection of OIIRR is crucial because it can be resolved once the orthodontic force ceases. This is to prevent the defect from becoming irreparable by cementum. Therefore, there is a need for establishing a sensitive and safer method to detect root resorption in the early stage and to be able to monitor the progression of root resorption during orthodontic treatment course.

Several studies have explored the potential of using dentine matrix protein as a specific biological marker for OIIRR [5–8]. Dentine sialophosphoprotein (DSPP) is one of the most abundant non-collagenous proteins in the dentine and it is highly dentine-specific because it was shown by earlier immunohistochemical studies to be only present in odontoblasts, predentine and dentine but not in other tissues or cells, such as enamel, bone, muscle, or cartilage [9, 10]. As compared to DSPP, the expression of other biomarkers such as inflammatory markers and bone remodelling markers may be affected by any other potential inflammation or bone metabolic problems in the body, making them less specific to OIIRR. The concentration of biomarkers in the gingival crevicular fluid (GCF) can be measured using enzyme-linked immunosorbent assay (ELISA). ELISA has been the gold standard method to detect a wide range of target molecules assisted with appropriate partner antibodies [11]. Despite being based on antigen-antibody reactions which offer high specificity and sensitivity, it is a labour-intensive method [12]. The test requires multiple tedious washing and incubation steps and, thus it is very time-consuming.

A prototype for root resorption detection was developed as a modification of the conventional ELISA method, using gold nanoparticles (AuNPs) as carriers for the capture antibodies and immobilising the conjugates onto wells of the microplate. Because of their large surface-to-volume ratio and high loading capacity, AuNPs provide an excess capture antibody binding sites for secondary antibody detection, resulting in a significant amplification of the colourimetric signal and greatly reducing the procedural time [13–15]. This enables the kit to be used at chairside. It yields rapid results in 45 min to 1 h compared to conventional ELISA which usually takes up 3.5 to 4 h to obtain its result. The intensity of the colour developed in the plate will be used to detect the presence of DSPP, by referring it to the colour intensity scale card provided in the kit. The concentration of DSPP in the GCF samples will be reflected by an increase in colour intensity. The use of this root resorption detection kit is relatively easy as it can be done by either dentist or dental surgery assistant.

The application of the root resorption detection kit seems promising for future monitoring of orthodontic management; therefore, this study aims to validate the use of this kit in apical root resorption detection by comparing it with the quantification of DSPP using a standard ELISA test and determine its user acceptance.

## Materials and methods

### Study design and ethical considerations

This was a cross-sectional study with ethical approval by the Research Ethical Committee of Universiti Kebangsaan Malaysia (UKM/PPI/111/8/JEP-2021-423). This study was conducted in accordance with the principles of the Declaration of Helsinki, and all patients provided written informed consent prior to enrolment. The sample size was calculated using G*power software version 3.1.9.7 downloaded from http://www.gpower.hhu.de/en.html. Based on the previous study done by Mah & Prasad, the effect size calculated from the means and standard deviation is 0.63 [8]. With the assumption that the two-sided significant level is 95 (1-alpha), a power of 95%, a total sample size of 45 subjects with 15 subjects in each experimental group is sufficient for this study.

Assessment of root resorption was done using periapical radiographs by one investigator (JHST) who had undergone calibration with RMAW, and the measurements were repeated after one month. The intra-class correlation coefficient test between the 2 sets of measurements was 0.84, showing a good repeatability of measurements. The measurements were done by using a digital vernier calliper (Tuten, USA) with a fine tip measuring ± 0.01 mm. The severity of apical root resorption detection by radiographic examination was referred to a scoring system introduced by Levander and Malmgren in 1988. They classified root resorption into 5 levels: 0 = no root resorption; 1 = mild resorption involving root with irregular contour; 2 = moderate resorption involving apical root resorption of less than 2 mm of original root length; 3 = severe root resorption involving apical root resorption from 2 mm to one third of original root length; 4 = extreme resorption with root resorption exceeding one third of original root length [16]. In this study, the above scoring system was adopted, and patients were divided into three groups: control (RO), mild (RM) and severe (RS). Group RO consisted of 15 untreated patients who have not started orthodontic treatment and with no radiographic evidence of root resorption (central incisors) as control; group RM consisted of 15 patients who were ongoing active orthodontic treatments with fixed appliances and there was radiographic evidence of mild root resorption of central incisors (< 2 mm); group RS consisted of 15 paediatric patients (8–11 years old) with the primary second molars undergoing active physiological root resorption and radiographic evidence of severe root resorption (≥ 2 mm) [8]. Paediatric patients were recruited because it is generally accepted by other researchers to use severely resorbed primary molars as the non-collagenous proteins in primary and permanent dentition are similar [5, 7, 8, 17]. The inclusion criteria for all the groups were patients with good general and periodontal health, no bleeding on probing, and no consumption of any anti-inflammatory drugs a month prior to the study. Patients with a previous history of orthodontic treatment and dental trauma were excluded from all the groups. The mean age of group RO was 22.07 ± 8.30, RM was 24.07 ± 2.71, and RS was 8.87 ± 0.92. Meanwhile, male subjects (*n* = 25, 55.6%) were slightly more than female subjects (*n* = 20, 54.4%).

### GCF collection and handling

Periopaper strip (Periopaper, Oraflow, Smithtown, N.Y.) was placed 1-2 mm into the gingival sulcus of the selected tooth for 60 s and this procedure was repeated for another two times with 1 min interval in between each collection. All three periopaper strips were immediately sealed into a 1.5 ml microcentrifuge tube containing 200μL of phosphate buffered saline to preserve the protein activity of the GCF. The above collection procedures were repeated twice and the samples were stored at − 80 °C before analyses using both ELISA and the root resorption detection kit [6, 8]. The investigator (JHST) was trained for the GCF collection by the clinical supervisors FY and RMAW.

### Newly developed root resorption detection kit

The patented newly developed root resorption detection kit was used according to the instructions given in the kit. All samples were assayed in duplicate. The colour intensity scale card provided in the kit was used by 2 examiners to categorize the severity of root resorption of the samples. Inter-rater reliability between 2 examiners was tested with Cohen’s kappa coefficient (κ). The kappa value (κ) was 1.0, indicating a perfect agreement between the 2 examiners (RMAW and FY). The optical density (OD) absorbance of the GCF samples on the microplate was obtained using a microplate reader (Varioskan Flash Multimode Reader, Thermo Fisher Scientific, Vantaa, Finland) at 450 nm.

### Enzyme-linked immunosorbent assay (ELISA)

Quantitative detection of DSPP in GCF was done using human DSPP ELISA kit (Wuhan Fine Biotech Co., Ltd, China). This kit uses sandwich ELISA technology with captured antibodies pre-coated onto a 96-well plate. All standards and samples were assayed in duplicate. A standard curve was constructed by plotting the mean OD of the samples (Y) versus the DSPP concentrations (X) of each standard and the best fit line was plotted using Microsoft Excel 2011. The concentration of DSPP (ng/mL) in the samples was then calculated by comparing the OD of the samples to the standard curve provided by the equation in the analysis.

### User acceptance questionnaire

To determine the acceptance of the newly developed root resorption detection kit by future users, a user acceptance questionnaire was adapted and modified from previous literatures [18, 19]. The questionnaire consisted of 13 questions, including 4 open-ended questions (1 main question and 3 sub-questions). For content validation, a panel of two experts was invited to evaluate the items. Some items in the questionnaire were rephrased to make it clearer and more relevant. Then, 10 subjects were recruited to pre-test the questionnaire. The reliability of the questionnaire was evaluated using Cronbach’s alpha. The calculated value of Cronbach’s alpha was 0.81, indicating the reliability of the questionnaire was good [20]. The validated questionnaire was then distributed to 30 orthodontists via Google Forms by email.

### Statistical analysis

The data analysis was done using SPSS version 26. Shapiro-Wilk test was used to test data normality. Sensitivity and specificity test was performed to evaluate the colour changes of different severity of root resorption measured using the root resorption detection kit. The sensitivity and specificity were calculated using the following equation:

$${\bf{Sensitivity}} = TP\,/\,(TP + FN) \times 100\%$$

$${\bf{Specificity}} = TN\,/\,(TN + FP) \times \,100\%$$

True positive (TP) is when the kit correctly detects root resorption in samples from RM and RS and true negative (TN) is when the kit correctly detects no root resorption in samples from group RO. While false negative (FN) is when the kit fails to detect root resorption and false positive (FP) is when the kit incorrectly identifies root resorption.

One-way ANOVA was performed to determine the statistical difference between the means of the DSPP and OD values in the 3 groups with various severity of root resorption. Pearson correlation coefficient was used to correlate the DSPP concentrations with the OD values, and the significance level was set at *p* < 0.05. The result from the questionnaire was analysed via descriptive analysis.

## Results

### Verification of the colour changes of different severity of root resorption with reference to the colour intensity scale card

**Fig. 1:**
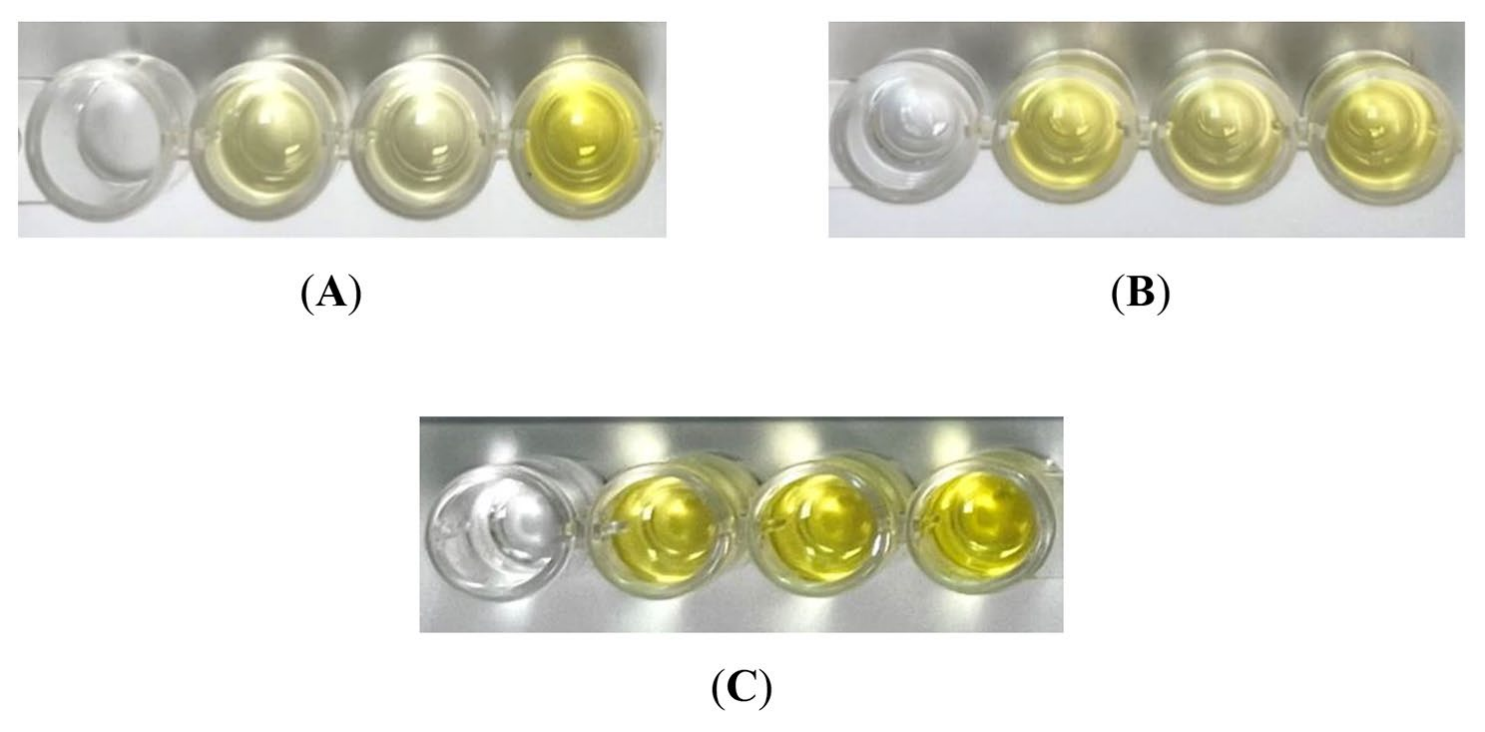
Difference in colour intensity formed by samples of different severity of root resorption: (**A**) control group (RO), (**B**) mild group (RM), (**C**) severe group (RS). The first well of the plate is the negative control well, the middle 2 wells are the sample wells, and the last well is the positive control well

Fig. 1 showed examples of pictures of the processed plate from the root resorption detection kit with different severity of root resorption. The sensitivity and specificity of the kit were 0.98 and 0.8 respectively (Table 1).

**Table 1:** Distribution of the results of the root resorption detection kit

	Clinical Conditions		Sensitivity	Specificity
**Kit Results**	**Presence of Root Resorption**	**No Root Resorption**	0.98	0.8
**Presence of Root Resorption**	(30 + 29)/2 True Positive (TP)	(2 + 4)/2 False Positive (FP)		
**No Root Resorption**	(0 + 1)/2 False Negative (FN)	(13 + 11)/2 True Negative (TN)		

*For a test to be useful, sensitivity + specificity should be at least 1.5 [21]

### Root resorption detection using ELISA

**Fig. 2:**
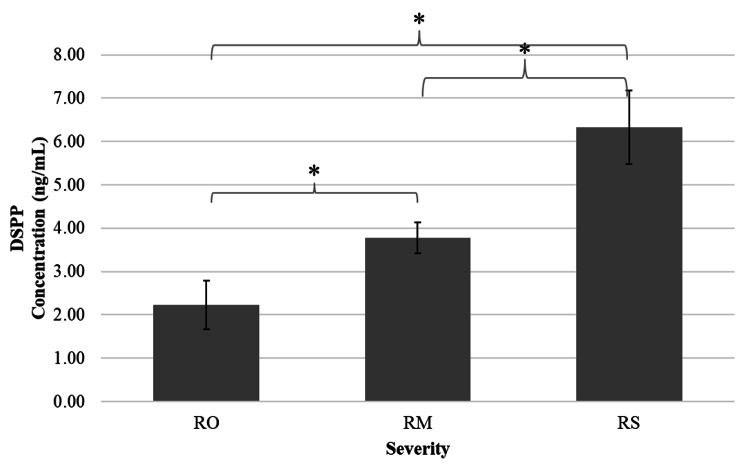
Comparison of DSPP concentration level in different severity of root resorption measured using ELISA method. *Significant (*p* < 0.05)

The DSPP concentration in GCF samples was the highest in the RS group (6.33 ± 0.85 ng/mL) while the RM group (3.77 ± 0.36 ng/mL) had less, and the RO group had the lowest concentration (2.23 ± 0.55 ng/mL) (Fig. 2). The one-way ANOVA analysis showed statistically significant differences in DSPP concentration among the three groups (*p* < 0.05). Post hoc analyses with Tukey’s HSD (using α of 0.05) revealed that all three groups showed statistically significant difference in DSPP concentration (*p* < 0.05).

### Root resorption detection using root resorption detection kit

**Fig. 3:**
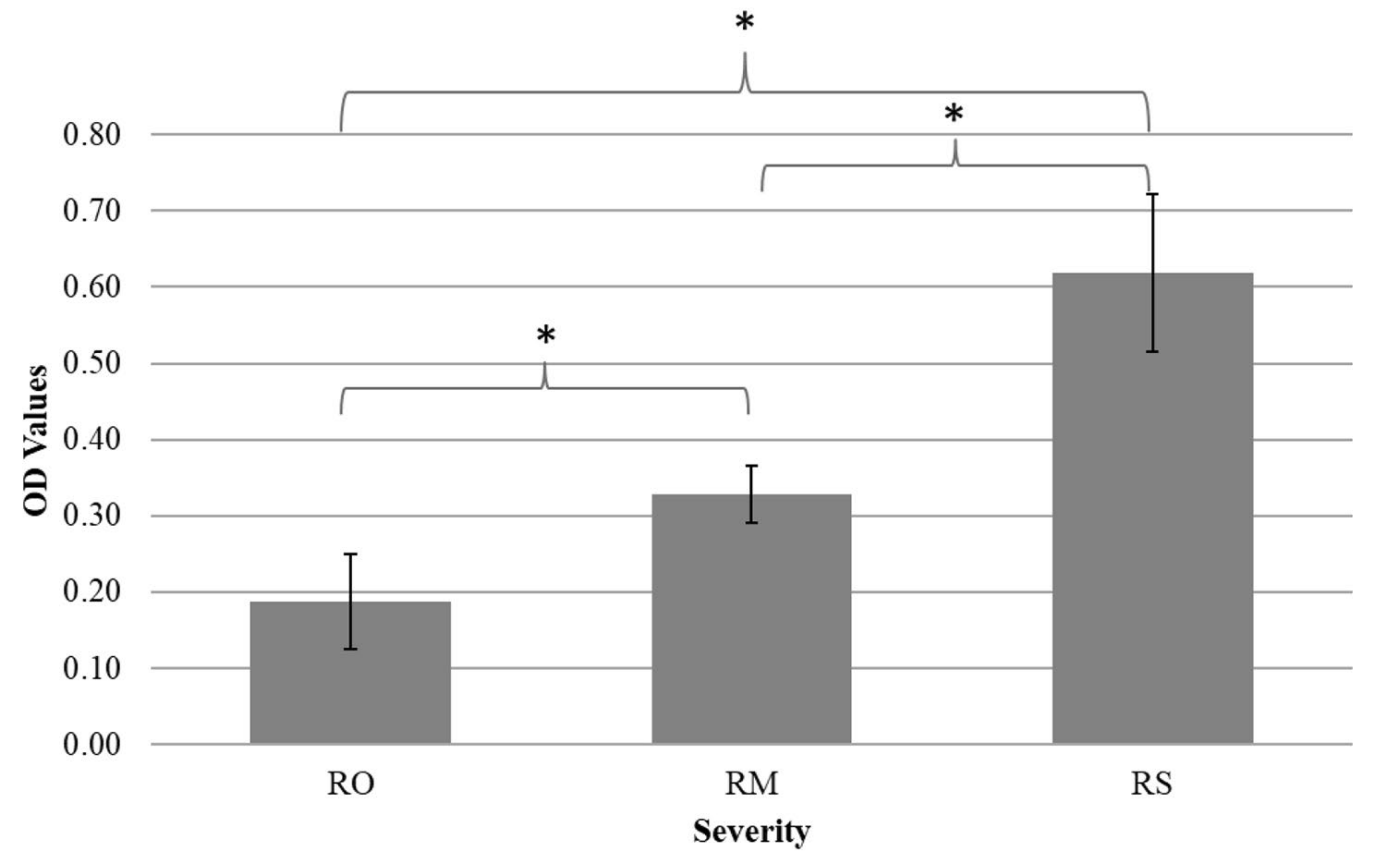
The OD values of different severity of root resorption measured using root resorption detection kit. *Significant (*p* < 0.05)

The different colour intensities formed using the root resorption detection kit were recorded in the form of OD values. The OD value was the highest in the RS group (0.62 ± 0.10) while the RM group (0.33 ± 0.03) had less, and the RO group had the lowest OD values (0.19 ± 0.06) (Fig. 3). The one-way ANOVA analysis showed statistically significant differences in OD values among the three groups (*p* < 0.05). Post hoc analyses with Tukey’s HSD (using α of 0.05) revealed that all three groups showed statistically significant difference in DSPP concentration (*p* < 0.05).

### Comparison of efficacy between root resorption detection kit and ELISA

**Fig. 4:**
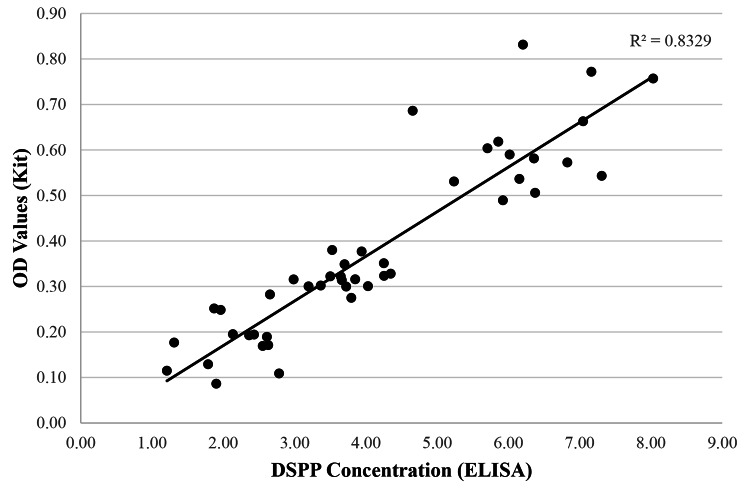
Scatterplot of OD values (Kit) against DSPP concentration (ng/mL) (ELISA)

Pearson correlation coefficient (Fig. 4) showed that there was a strong and positive correlation between DSPP concentration and OD values, which was statistically significant (*r* = 0.913, *p* < 0.001, *N* = 45). The OD values increased linearly with the increase in DSPP concentrations.

### User acceptance questionnaire

A total of 30 orthodontists (7 males, 23 females) completed the user acceptance survey for the root resorption detection kit. The mean age was 40.13 ± 4.89-years-old. Most of the respondents worked in the government sector (73.3%) and do not own a dental clinic (93.3%). Table 2 shows the summary of the responses from the questionnaire.

**Table 2:** Summary of identified themes and responses

Themes	Responses					
**Root resorption experience**						
	**Often**	**Sometimes**			**Rarely**	
1. Frequency	6.7%	60.0%			33.3%	
2. Standard method to detect root resorption	Periapical radiographs, orthopantomography, cone-beam computed tomography					
	**Yes**			**No**		
3. Ever heard of using biomarkers to detect root resorption?	40.0%			60.0%		
**Opinions on root resorption detection kit**						
	**Below average**		**Average**			**Above average**
1. Usefulness	3.3%		46.7%			50.0%
2. Value for the money if the kit is RM50/testing	13.3%		63.3%			23.3%
3. Efficiency of the kit if the kit could yield results within an hour	20.0%		43.4%			36.7%
4. Colour detection method	0%		16.7%			83.3%
**Acceptance of the kit**						
	**Not likely**		**Neutral**			**Likely**
1. Willingness to buy the kit	10.0%		43.4%			46.6%
2. Likeliness of the kit to replace or supplement the current method of detecting root resorption	20.0%		40.0%			40.0%
3. Likeliness to recommend to friends or colleagues	3.3%		46.7%			50.0%
4. Likeliness to participate in the clinical testing process	30.0		-			70.0%

## Discussion

OIIRR is known to be a common sequela of orthodontic treatment, hence, early diagnosis is crucial as it can help orthodontists to modify their treatment plans accordingly. DSPP has been proven to be a reliable biomarker for OIIRR by previous researchers [17, 22, 23]. In their studies, ELISA was used in combination with spectroscopy to quantify the concentrations of the DSPP. However, it is impractical to use it routinely to detect or monitor the OIIRR as it takes long hours to do the assay. The root resorption detection kit was a modified version of conventional ELISA by adding gold nanoparticles (AuNPs) as a carrier for the primary antibodies (anti-DSPP) to overcome the limitations of ELISA. These AuNPs provide excess binding sites for secondary antibody (anti-DSPP-HRP) detection, resulting in a significant amplification of the colourimetric signal and a reduction in the procedural time. By referring to the colour scale card that is provided in the kit, the intensity of the colour formed from the samples indicated the severity of the OIIRR.

In this study, the efficacy of the root resorption detection kit was compared to a conventional ELISA in detecting DSPP in the GCF among 3 groups of patients with different severity of root resorption. A group of subjects aged 8–11 years old with their upper primary second molars undergoing physiological root resorption was recruited to the severe group. This is due to difficulty to recruit orthodontic subjects with significantly severe root resorption, as the prevalence is very low [17]. The molecular content of the non-collagenous proteins in primary and permanent dentition is similar, hence, the use of physiological root resorption is generally accepted by various researchers to apply to the study of pathological root resorption [5, 7, 8]. Although there are microscopic anatomical differences and the initiation process of root resorption that may vary between primary and secondary dentitions, the biochemical mechanism that occurs is very much similar [7, 17].

In terms of sensitivity, the kit correctly detected 98% of the samples with root resorption, which were the samples taken from the RM and RS groups. Whereas for specificity, the kit correctly detected 80% of the samples without root resorption, which were the samples taken from the controls. This showed that the colour formed in the plate can be easily differentiated by the users to detect the presence of root resorption. Since the developed detection kit was the first of its kind to detect OIIRR, no comparison of a similar tool can be made. The colour formation in the RO group indicated that the DSPP was present in the samples even though these teeth demonstrated no signs of root resorption from radiographic evidence. This finding was in accordance with the results from previous studies [4, 5, 7, 8]. This might be due to the antibodies used in the study being polyclonal antibodies that might have reactions with proteins of the same isotopes. Another plausible explanation for the expression of DSPP in the RO group could be due to minor structural changes during physiological root resorption. Histologically, root resorption affects even untreated teeth, particularly at the apical root region [4]. Collectively, these explanations taken together may help to explain the expression of DSPP in the RO group.

The concentration of DSPP was quantified in relation to the severity of root resorption using ELISA in this study. The DSPP concentration level was found to be highest in the RS group, followed by RM and RO. These results are in good agreement with the previous studies [4, 5, 7, 8]. Meanwhile, the optical density (OD) values were recorded according to the different colour intensities formed using the root resorption detection kit. Similar to the ELISA method, the root resorption detection kit was able to distinguish the severity of root resorptions among all the samples. The highest DSPP concentration in the RS group was anticipated as physiologically resorbing upper first deciduous molars involve extensive and complex root resorption. The samples in the RM group were taken from upper permanent incisors with radiographic evidence of mild root resorptions, which was relatively lesser than the RS group, thus the lesser amount of DSPP detected in the GCF [8]. On the other hand, Pearson correlation coefficient indicated that there was a strong and positive correlation between DSPP concentration and OD values, which was statistically significant (*p* < 0.001). This demonstrated that the root resorption detection kit can be used to detect different severity of root resorptions on par with that of the current gold standard method (ELISA).

A survey to determine the acceptance of future users. From the survey, all the respondents reported having encountered root resorption in their daily practice. This is in line with the fact that OIIRR is generally agreed to be an unavoidable sequela of orthodontic treatment [24–26]. Periapical radiograph was the most frequently used method to detect root resorption due to it having lesser image distortion and being able to provide necessary details with lower radiation exposure to the patients compared to the orthopantomogram [27]. Nevertheless, orthopantomogram was quite popular among orthodontists to use as a tool to detect root resorption because it is the standard radiograph required before starting orthodontic treatment [1]. On the other hand, most of the respondents (60.0%) never heard of using biomarkers to detect root resorption. This is acceptable given that the approach is still mostly utilised for research purposes and there is not currently a detection kit available on the market specifically developed to detect root resorption at the chairside. The second theme of the questionnaire was about the opinions on the root resorption detection kit. Generally, the respondents were satisfied with using colour intensity to identify the severity of root resorption. However, some respondents suggested the price should be lower to Malaysian Ringgit 10–25 per test and the time to yield the result to be shortened to within 15 min. In fact, the root resorption detection kit has greatly reduced the time needed to detect the DSPP biomarker from 3.5 to 4 h using the conventional ELISA to around an hour. Nevertheless, further research is required to develop a more practical detection kit for clinical use. After all, most of the replies were neutral-to-favourable when it came to the acceptance of the kit. According to the results of the survey, the root resorption detection kit would be able to meet the respondents’ expectations of being a safer, simpler, and more reliable approach for root resorption detection.

Some of the previously reported limitations were encountered in this study as well. The assessment of root resorption severity was based on the existing conventional 2D radiographs which may have some degree of error [5, 8]. It would be suggested that future studies can be conducted utilising 3D digital radiology like cone beam computed tomography (CBCT), which has a more accurate assessment of the severity of root resorption. However, routine use of the CBCT for orthodontic patients is not indicated. The prescription of a CBCT should be based on robust evaluations of a risk to benefit ratio for the patient. Besides, it is ethically unjustified to subject patients to unnecessary radiation doses of CBCT solely for research purposes.

Another limitation of this study was the small sample size. Although the minimal requirement for a sample size of 45 subjects was met to detect any significant difference in DSPP concentration among the different severity of root resorption, a greater number of samples could be included in the future study to further improve the power of the study.

## Conclusion

The findings of this study demonstrated that the newly developed root resorption detection kit was able to display differentiable colour intensities by detecting the presence of DSPP in the GCF. The colour intensity was greatest in the RS group, followed by the RM group, and lowest in the RO group comparable to outcomes obtained using the gold standard, ELISA. This showed that the root resorption detection kit is just as efficient as ELISA in detecting DSPP in the GCF, with the added value of chairside clinical diagnostic. Future users find that the kit is a simpler, no radiation exposure, less time-consuming and sensitive alternative for early detection of OIIRR.

## Data Availability

The datasets used and/or analysed during the current study are available from the corresponding author on reasonable request.
